# A laboratory preparation procedure for studying bioaccumulation of U and its subcellular form in earthworms (*Diplocardia* spp.)

**DOI:** 10.1016/j.mex.2022.101755

**Published:** 2022-06-10

**Authors:** Lanre Olafuyi, Naira Ibrahim, Jing Nie, Precious Cooper, Steven L. Larson, John H. Ballard, Ahmet Celik, Shaloam Dasari, Saiful M. Islam, Fengxiang X. Han

**Affiliations:** a Department of Chemistry and Biochemistry, Jackson State University, Jackson, MS 39217, USA; b U.S. Army Engineer Research and Development Center, Vicksburg, MS 39180-6199, USA; c Department of Biology, Jackson State University, Jackson, MS 39217, USA

**Keywords:** Earthworm, Uranium, Subcellular Distribution, Bioaccumulation

## Abstract

Uranium (U) is a ubiquitous trace element in soils. With increasing in application of U in nuclear energy and nuclear weapon, a large amount of U was dissipated into the environment including soil and water. Earthworm may be an eco-indicator for U bioaccumulation, transformation and transport across the ecosystem. There have been a variety of methods preformed to assess the bioaccumulation of uranium in small organisms such as earthworms, including uranium speciation, subcellular separation, and total U accumulation. All methods require an initial grinding preparation process that allows for the further fractionation of metals and metalloids in earthworms. The slime like mucus that coats the body of a worm presents a challenge in the disintegration and dissolution of the worm body. In order to analyze U subcellular forms, we developed a reliable and effective procedure to grind the worm body into a uniform fine suspension. We conducted a comparative study of disintegration of worms with 3 grinding techniques (agate mortar, liquid nitrogen freezing then agate mortar, and direct sonication) that would assist U subcellular analyses and bioaccumulation. The essences of this new development was as follows:

• A scheme for preparation of earthworm samples for investigation of subcellular U forms in earthworms from U.S. army weapon test range soil with various U forms.
• The direct sonication of earthworms was found to be the most proficient process in achieving the best preparation for U subcellular analyses with the high precision.

## Graphical abstract

**Figure:**
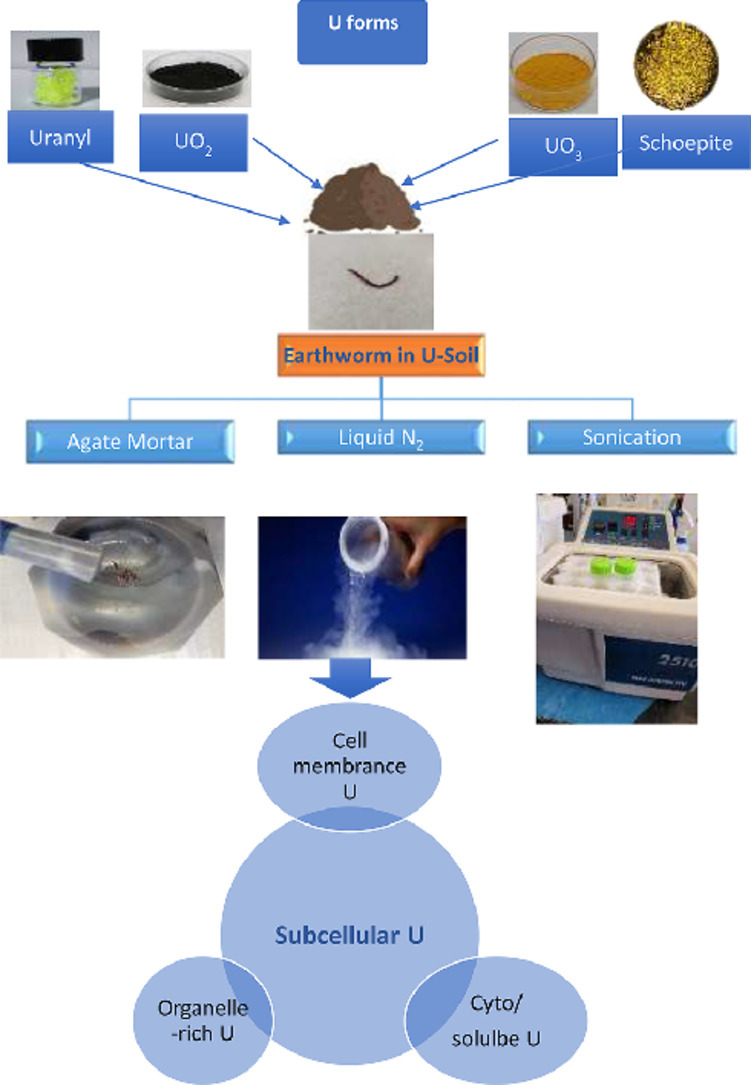

**Specifications table**

**Table:** 

Subject Area;	*Environmental Science*
More specific subject area;	*Heavy metal pollution control and remediation*
Method name;	A laboratory preparation procedure for studying bioaccumulation of U and its subcellular form in earthworms
Name and reference of original method;	*X. Nie, F. Dong, N. Liu, M. Liu, W. Zhang, S. Sun, J. Yang. (2014)* An investigation on the subcellular distribution and compartmentalization of uranium in Phaseolus vulgaris L. *J Radioanal Nucl Chem (2014) 299:1351–1357.*
Resource availability;	*N/A*

## Method details

### Background

Uranium (U), a natural trace element in earth crust, has been released into the environment through anthropogenic activities such as mining and processing, industrialization, nuclear power plants, and nuclear manufacturing as well as application of depleted uranium products. Uranium concentration is in a range of 0.3-11.7 mg kg^−1^ in rocks and soils with an average of 3 mg kg^−1^ [1,2]. Three main uranium isotopes (U238, U235 and U234) are all radioactive.

Depleted uranium (DU) is the by-product of the U enrichment process. DU was used in military applications such as armor-piercing projectiles known penetrators and for armor-plating tanks based on its pyrophoric properties and high density. Several hundred tons of DU were released in military conflicts over the past forty years and DU was expected to remain in battlefields and army testing sites [3], [4], [5], [6], [7]. We previously reported that significant U was accumulated in army weapon test sites – Yuma Proving Ground site [5], [6], [7]. In the fields of Yuma Proving Ground, 92.8% of U from DU penetrators and fragments remained in the top 5 cm of soil and decreased to background concentrations in less than 20 cm [7]. In locations prone to high amounts of water runoff, U concentrations were reduced significantly after 20 m from the source due to high surface runoff [7]. U was also transported throughout the ecosystem via plant uptake and wild animal consumption between trophic levels, but with limited accumulation in edible portions in plants and some animals [7].

Uranium poses multiple health risks, especially chemical toxicity. DU is a low radioactive α-emitter material, therefore acute risk is not likely from external exposure. However, the potential hazard is a result from internal exposure, radiological and chemical effects [8,9]. Uranium from embedded DU fragments may redistribute resulting in high levels of several oncogenes that are associated with carcinogenesis [9].

Earthworms are an essential family of soil biomass and are viewed as a valuable marker of soil condition and quality. Earthworms are the environment's natural decomposers and they survive under a variety of habitat conditions that make them viable candidates for environment monitors [10]. Earthworms can survive and reproduce in various places with concentrated pollution and can also accumulate metals such as Hg and Cd in its body [11,12]. The earthworm species *Lumbricus terrestris* was known as knight crawlers originated in Western Europe but became common species in North America through trade and immigration.

Bioaccumulation is the accumulation of hazardous chemicals in any living organism such as plants, animal insects or other microorganisms. The longer the toxins remain present in the organisms, the stronger the effects of the toxins become regardless of the region having low hazardous chemical concentrations. Earthworm was used for bioindicators for many contaminants in soils such as Hg [12] and U in soils [13]. However, uniform grinding earthworms proved challenging due to the soft slime like mucus that coats their body [11]. The objective of this study was to develop the effective grinding procedure to disintegrate earthworms into a homogeneous suspension for studying subcellular U forms in earthworms.

The methods we evaluated consisted of 1) Direct agate mortar grinding of fresh samples, 2) Liquid nitrogen freezing – agate mortar grinding, and 3) Sonication disintegration of the worms into the suspension solutions.

Procedures:

1. Soil sampling and preparation.

1.1. Soil samples were taken from Riley military base, KS at the depth 0–30 cm. The soil was sieved to remove particles greater than 2.00 mm.
1.2. To produce simulated uranium contaminated soil for the control soil type, uranium schoepite (<250 µm) was added to produce U contaminated soil that contained 6,000 ppm uranium delivered through aging at 55°C for sixty days. For the dry spiking, schoepite (<250 µm) was mixed with 500 g dry soil in mixing pan and was mixed for 1 min. The soil sample was then passed through a sample splitter for a total of twelve times to ensure homogeneity. The remainder soil (total 4 kg soil) was mixed as mentioned above and added and mixed for 8 × 1-min intervals. The spiked soils (4 kg) were placed in open plastic buckets for artificial aging in the environmental chamber at 55 degrees C. The samples were maintained at the water holding capacity (WHC) of 50% throughout the 60-day time period.

2. Earthworm incubation in U spiked soils

2.1. About 0.5 kg of farm raised North American night crawlers (*Diplocardia* spp.) were purchased from a local community garden and were transported in generic pot soil and worm casting.
2.2. About 20-30 worms weighing at 2-3 grams were lightly washed with warm water.
2.3. Worms were placed into the middle of the U soils to ensure the maximum exposure.
2.4. Worms were exposed to the soils for 35 days. After 35 days of exposure worms were lightly cleaned and prepaired for purging (no worm casting was added to soil).

3. Worm purging preparation

3.1. After the initial soil exposure, three worms were extracted from each soil sample. Two layers of wet filter paper were placed into a petri dish with the worm samples.
3.2. The worms were left for 48 hours to purge while keeping a consistently moist filter paper present.
3.3. After purging worms were cleaned and were cut into smaller pieces using an exact knife and were placed aside for later use.

4. Three methods were used to prepare the worms for U study: Agate mortar grinding technique, liquid nitrogen freezing then grinding, and direct sonication.
5. Preparation Method 1: Agate mortar grinding technique with fresh earthworms

5.1. Sections of three fresh purged worms (around 0.2 g) were extracted from each contaminated soil sample and cut into micro beads.
5.2. The worms were then transferred to the well of the mortar and grinded to the finest form attainable.
5.3. The worm was then placed in a 50 ml centrifuge tube and homogenized with the given solution and prepaired for further U subcellular study.

6. Preparation Method 2: Liquid nitrogen freezing and grinding with agate mortars

6.1. Sections of three fresh purged worms (around 0.2 g) were extracted from each contaminated soil sample and cut into micro beads.
6.2. The worm samples were then frozen in liquid nitrogen for 24 hours. After freezing the samples were transferred to aluminum container and submerged in liquid nitrogen. As quick as possible, the sample was grinded with a metal mallet till the sample was in a fine form.
6.3. The samples were transferred to a 50 ml centrifuge tube and homogenized with the given solution and prepared for further U subcellular study.

7. Preparation Method 3: Direct sonication technique

7.1. Sections of three fresh purged worms (around 0.2 g) were extracted from each contaminated soil sample and cut into micro beads.
7.2. The worms were then transferred to a 50 ml centrifuge tube with 1-2 ml water and placed in the sonicator for three hours or until the solid sample turned to liquid.
7.3. The samples were then homogenized with the given solution and prepared for further U subcellular study.

8. Subcellular U forms in earthworms [14]

8.1. Three subcellular U forms/ fractions were determined. Sections of three fresh worms (around 0.2 g) were prepared from three different preparation methods Steps 5-7.
8.2. The prepared suspensions were placed in a 50 ml centrifuge tubes and its volume was made to 10 ml of the total suspension.
8.3. The total volume (10 ml) of the suspension was divided into two equal portions (5 ml). The first portion with 5 ml of the mixture was used to determine the total U in the earthworm. A second 5 ml supsension was used for determinng the subcellular U forms.
8.4. One litter of a homogenized buffer solution was made using 0.0315 grams of tris HCL, 0.17419 grams of phenylmethane sulfanyl fluoride, 0.37224 grams of EDTA and 0.28838 grams of sodium dodecyl sulfate. The pH of the buffer solution was adjusted to 8.0 using HNO_3_ and ammonium hydroxide.
8.5. The buffer solution was added to the earthworm sample (5 ml suspension) until volume of the sample reached 15 ml. Samples were shaken for 10 minutes then centrifuged at 500 rpms for 5 minutes.
8.6. The pellet was considered as the cell membrane. The pellet was digested with 0.5 ml of nitric acid and 10 drops of 30% H_2_O_2_ on the hotplate until the solid was completely desolved. The digestion volume was brought up 10 ml then filtered with a 0.45 µm filter for inductively coupled plasma optical emission spectroscopy (ICP-OES) anaylsis.
8.7. The supernatant was brought up to 15 ml with water and centrifuged at 10,000 rpms for 30 minutes. The resultant supernatant solution was considered to be the cytosol fraction, including macromolecular organic matter and inorganic ions in the cytoplasm and vacuoles [14]. The micro pelets remaing was considered as the organelle fraction which was further digested with 0.5 ml of nitric acid and 10 drops of 30% H_2_O_2_ until the solid residual was completely dissolved. The sample was brought up to 10 ml with water and filtered with a 0.45 µm filter for ICP analysis.

9. Acid digestion of earthworms for the total uranium analysis

9.1. A portion of fresh worm (5 ml) from Step 8.3. was placed into a 50 ml centrifuge tubes.
9.2. Two ml of HNO_3_ and 2 ml of 30%H_2_O_2_ were added to the mixture and additional H_2_O_2_ was added as needed until all tissues were digested.
9.3. After digestion the supernatant was filtered and set aside for ICPOES analysis.

10. U in the filtered solution was determined with ICP mass spectroscopy (ICPMS) or ICPOES.

## Method validation

**Fig. 1:**
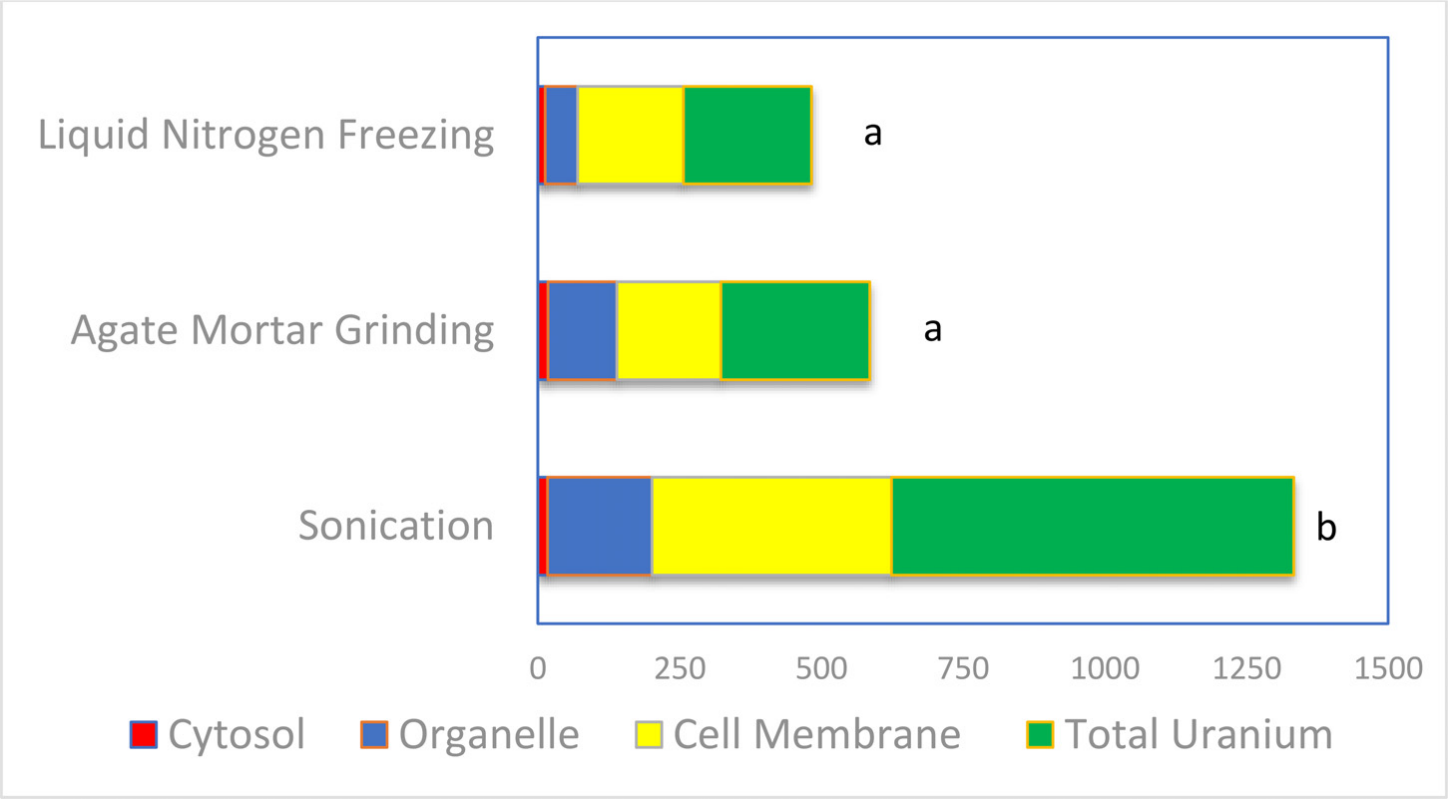
Comparison of total U and U subcellular forms in earthworms prepared with 3 methods (Liquid N_2_ freezing, Agate mortar grinding, and Sonication). Earthworms grew in U contaminated soil. U in earthworms was divided as the Cytosol, Organelle, and Cell membrane bound U fractions and total U. The different letters indicate the significant difference among the methods of preparation.

**Fig. 2:**
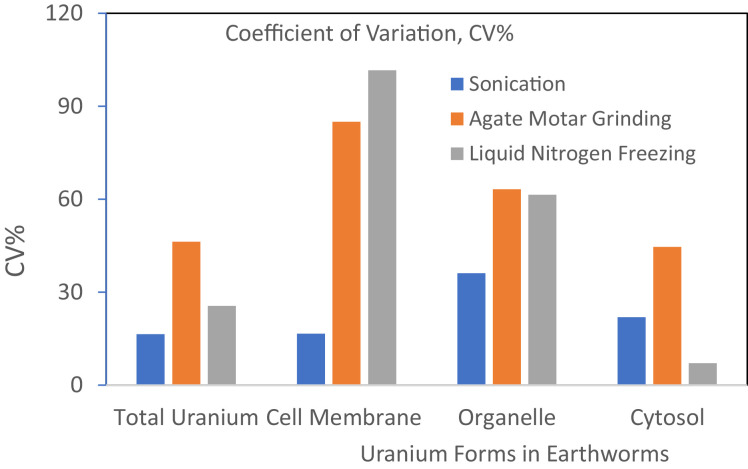
Comparison of coefficients of variations (CV%) of U in subcellular forms and total U in earthworms, prepared with different methods.

Fig. 1 depicts the concentrations of total uranium and subcellular U forms present in earthworms from U contaminated soil. The sonication method for preparation of earthworm samples showed the highest total U concentrations and highest U concentrations in all subcellular forms (Cytosol, Organelle, and Cell membrane fractions) (Fig. 1). The agate mortar grinding method produced the second highest concentrations in total U and subcellular U forms. However, liquid N_2_ freezing following by agate mortar grinding generated the lowest total U and U subcellular forms. This implies that direct sonication method kept all original U without any potential loss during the preparation. Further, sonication method had the lowest coefficient of variation (CV%) between replications, while agate mortar grinding and liquid N_2_ freezing followed by grinding both had the much higher CV% (Fig. 2). This indicates that sonication preparation method gave a higher precision of measurements of total U and subcellular U forms compared other two conventional direct grinding of earthworm samples. Thus, the current study proved that direct sonication method was the best rapid laboratory procedure to prepare earthworm samples for studying bioaccumulation of U, other heavy metals and trace elements in earthworms.

## Declaration of Competing Interests

The authors declared no known competing financial interests or personal relationships that could have appeared to influence the work reported in this paper.

## Data Availability

Data will be made available on request.
